# Tolerability of Daratumumab Amongst Asian Patients with Plasma Cell Dyscrasias – A Single Centre Experience.

**DOI:** 10.46989/001c.92085

**Published:** 2024-01-16

**Authors:** Allison C. Y. Tso, Samuel S. Y. Wang, Christian A. Gallardo, Dheepa Christopher, Kiat Hoe Ong

**Affiliations:** 1 Haematology Tan Tock Seng Hospital

**Keywords:** myeloma, daratumumab, CD38, monoclonal antibody, asian

## Abstract

Daratumumab is increasingly incorporated into the standard treatment regimens for patients with plasma cell dyscrasias in Asia, especially with cost-containment measures implemented by various regional health authorities. This analysis aimed to study daratumumab’s tolerability amongst Asian patients. This is a retrospective medical records review of patients who received daratumumab between November 2016 and August 2021 as part of routine clinical care. Sixty-two patients were included in the study: 62.9% had renal impairment, and 27.4% had creatinine clearance (CrCl) <30ml/min. Forty-five patients (72.6%) received daratumumab combination therapy, with a median 1 line of prior therapy. The median duration of follow-up was 12.4 months, and the median duration patients were on treatment with daratumumab was 12.3 months. Twenty-one of 62 (33.9%) patients experienced infusion-related reactions (IRRs) after the first dose of intravenous daratumumab. Seven developed mostly grades 1 and 2 respiratory events, and 14 showed grades 1 and 2 non-respiratory IRRs. Only one patient experienced a grade 1 IRR with the second infusion, with none developing any IRRs in the third or subsequent infusions. Eight (12.9%) patients were affected by hematological adverse events (AEs), mostly grades 2 and 3, with one experiencing grade-4 neutropenia without sepsis. Six (9.7%) patients experienced non-hematological AEs, the commonest being pneumonia and other infections, with one developing Nocardia pneumonia (grade 4) 14 months after the initiation of daratumumab. In conclusion, daratumumab is tolerable amongst Asian patients, including the elderly, and patients with severe renal impairment and chronic lung diseases.

## Introduction

Daratumumab is a human CD38 IgGk monoclonal antibody that targets and eliminates myeloma cells via three main immune-mediated mechanisms, including complement-dependent cytotoxicity (CDC), antibody-dependent cellular cytotoxicity (ADCC)^1^ and antibody-dependent cellular phagocytosis (ADCP).^2^ It also modulates the tumor microenvironment to overcome conventional drug resistance, and it has a direct anti-tumor effect through apoptosis after cross-linking.^3^

Since daratumumab monotherapy was granted FDA approval in November 2015 for heavily pre-treated patients with relapsed refractory multiple myeloma, it has been approved for use in combination with other myeloma agents, in earlier lines of therapies, including in newly diagnosed transplant-eligible and ineligible patients. It is also approved for the treatment of newly diagnosed light chain (AL) amyloidosis based on the ANDROMEDA (NCT03201965) study.^4^

Daratumumab is generally well tolerated. In the GEN501 and SIRIUS combined dataset, the rate of all infusion-related reactions (IRR) grades was 48%.^5^ In the CASTOR study,^6^ the IRRs were 45%, with 9% grade 3, and no grades 4 or 5. The rate of IRRs in the POLLUX study^7^ was 48%, with 5% grade 3, and no grades 4 or 5. All grades of treatment-emergent adverse events (TEAEs) ranged from 14% to 59%.^5–7^ Clinical trials often exclude patients with severe renal impairment (CrCl <30mL/min); Asian subgroups are often under-represented. Likewise, real-world experience on the tolerability of daratumumab in these patient subgroups is also limited.

The time taken from marketing authorization for novel therapeutics to inclusion into the national drug formulary outside of the US and EU can be considerable. In Singapore, daratumumab monotherapy was approved by the Health Science Authority (HSA) in October 2016, and since October 2017, combined with bortezomib or lenalidomide, for treating relapsed refractory multiple myeloma patients. Since 2020, daratumumab combination therapy has been approved for use in the front-line setting in both transplant-ineligible (daratumumab, lenalidomide, and dexamethasone) and transplant-eligible (daratumumab, bortezomib, thalidomide, and dexamethasone) multiple myeloma patients. The indication for daratumumab in combination with bortezomib, cyclophosphamide, and dexamethasone was extended to newly diagnosed patients with light-chain AL amyloidosis in February 2022.

As daratumumab gains more clinical indications (e.g., newly diagnosed AL amyloidosis), with rigorous cost containment measures^8^ and national reimbursement drug list negotiations,^9,10^ and with the development of biosimilar HLX-15 (NCT05679258, estimated primary completion December 2023),^11^ there will likely be a significant increase in patient access to daratumumab in markets outside of the US and EU, in the foreseeable future.

As Singapore is one of the first countries where daratumumab was launched, this drug has been incorporated in the routine treatment of patients with newly diagnosed and relapsed/refractory multiple myeloma and other plasma cell dyscrasias for some time, ahead of other countries in the region. We are sharing the results of this real-world observational study on the tolerability of intravenous (IV) daratumumab amongst Asian patients.

## Methodology

This is a retrospective single-center study of patients who received IV daratumumab between November 2016 and August 2021 as part of routine clinical practice. The study is approved by the National Healthcare Group (NHG) Institutional Review Board ethics committee (2020/01305). Patient demographics, disease characteristics, prior treatment, complete blood count (FBC), renal and liver function prior to commencement of daratumumab, pre and post infusion medications, and IRRs, were recorded from the medical notes. All hematological and non-hematological adverse events (AEs) that were documented in the electronic patient medical records from the date of initiation of daratumumab up until their last follow-up, based on the cut-off date of the study, were recorded, regardless of whether the events were attributed by daratumumab therapy. Grading of reactions was according to the Common Terminology Criteria for Adverse Events (CTCAE) version 5.0. The data cut-off was set as 30^th^ September 2021.

## Results

The median duration of follow-up was 12.4 months (range 0.6 - 41.4 months). A total of 62 patients (35 males and 27 females) were included in the study (**Table 1**). Their median age was 67.5 years (range 30 - 89). Twenty-seven (43.5%) were aged <65 years, 21 (33.9%) were aged 65 - 74 years, and 14 (22.6%) aged ≥75. Their weight ranged from 35.2 to 83.2kg (median 58.7kg). The majority (n=44) of patients were ECOG 0, 13 were ECOG 1, two ECOG 2, and one ECOG 3. Six of the 62 patients had underlying asthma or chronic lung disease. Most (n=57) of the patients had a diagnosis of multiple myeloma, 3 had AL amyloidosis and 2 had other plasma cell dyscrasias (POEMS syndrome and monoclonal gammopathy of renal significance (MGRS) respectively).

**Table 1.** Patient demographics, disease characteristics and prior therapies.

**Patient Characteristics**			
Gender		Diagnosis	
Male	35	Myeloma	57
Female	27	AL amyloidosis	3
Race		Other plasma cell dyscrasias	2
Chinese	42	Heavy Chains	
Malay	15	IgG	32
Indian	5	IgA	12
Age		IgD	1
<65	27	No heavy chains	15
65-74	21	Unknown	2
≥ 75	14	Light chains	
ECOG		Kappa light chains	36
0	44	Lambda light chains	23
1	13	Unknown	3
2	2	ISS Staging for myeloma patients	(n=57)
3	1	I	4
Unknown ECOG	2	II	20
Underlying lung disease		III	29
Yes	6	Unknown	4
No	56	RISS Staging for myeloma patients	(n=57)
Renal Function		I	2
CrCl >60mL/min	23	II	27
CrCl 30-60 mL/min	22	III	20
CrCl <30mL/min	17	Unknown	8
Liver Function		FISH	
Normal ALT, AST	60	High Risk*	22
Abnormal ALT, AST	2	Non high risk	31
		Unknown	4
**Treatment History**			
Prior Stem cell Transplant		Prior Proteosome Inhibitors (PIs)	
Yes	8	Yes	41
No	48	No	15
Unknown	6	Unknown	6
Prior Lines of Therapy		Prior IMIDs	
0	16	Yes	25
1	25	No	31
2	8	Unknown	6
3	4	Prior PI and IMIDs	24
4	2	Treatment regimens	
5	1	Daratumumab combination therapy	45
Unknown	6	Daratumumab monotherapy	11
		Unknown	6

*high risk FISH (Fluorescence in situ hybridization) include t(4;14)(p16;q32), t(14;16)(q32;q23), 17p13 deletions and chromosome 1 abnormalities.

Over half (50.9%) of the 57 multiple myeloma patients belonged to ISS stage III (n=29) and over a third (35.1%) belonged to the revised ISS (RISS) stage III (n=20). A total of 38.6% of patients (n=22) had high-risk FISH (fluorescence in situ hybridization), defined by the presence of t(4;14)(p16;q32), t(14;16)(q32;q23), 17p13 deletions and aberrations of chromosome 1. Thirty-one myeloma patients had non-high-risk FISH. The FISH results of 2 myeloma patients were not available. The 3 patients with AL amyloidosis and the 2 with other plasma cell dyscrasias had no high-risk FISH features.

### Laboratory Findings

Twenty-three (37.1%) patients had normal renal function defined by CrCl of >60mL/min. Thirty-nine (62.9%) had renal impairment defined by CrCl <60mL/min, and 17 (27.4%) had CrCl <30ml/min, an exclusion criterion commonly applied in clinical trials. Two of the 17 patients with a CrCl of <30ml/min were initiated on hemodialysis before daratumumab therapy. The rest did not require hemodialysis support. Sixty of 62 patients had normal liver function tests, defined by normal aspartate aminotransferase (AST) and alanine transaminase (ALT). The cohort’s hemoglobin (Hb) ranged from 6 - 14.8g/dL (median 9g/dL); absolute neutrophil count (ANC) ranged from 0.7 - 12.9 x10^9^/L (median 3.38 x10^9^/L); and the platelet count ranged from 28 - 642 x10^9^/L (median 174.5 x10^9^/L).

### Previous Therapies

There were altogether 16 newly diagnosed patients in the study. One of them received daratumumab-based induction therapy, underwent an autologous stem cell transplant, and continued with daratumumab-based therapy. The prior treatment records from 6 patients were not known. Among the 40 patients who received at least 1 prior line of therapy, 39 were previously treated with a proteasome inhibitor (PI), and 24 with an immunomodulatory drug (IMID). Twenty-three patients were exposed to both classes of drugs. Excluding the 2 patients with other plasma cell dyscrasias (POEMS syndrome and MGRS), only 7 (18.4%) of the 38 patients received a prior autologous stem cell transplant, although 18 (47.3%) were aged <65 years. Amongst these 18 patients aged <65 years, 2 had a diagnosis of AL amyloidosis, and 16 had myeloma. Nine were Chinese, 7 were Malay, and 2 were of Indian origin. Although we routinely refer all patients aged <65 for consideration of autologous stem cell transplantation, the relatively low uptake may be explained by fitness considerations, reimbursement and financial concerns, and the tendency for risk aversion amongst the local population.

Among previously treated patients, the median line of prior therapy was 1. In the entire cohort, 45 patients (72.6%) received daratumumab combination therapy: DRd (daratumumab, lenalidomide and dexamethasone) (n=24), DVd (daratumumab, bortezomib and dexamethasone) (n=11), DVTd (daratumumab, bortezomib, thalidomide and dexamethasone (n=3), DPd (daratumumab, pomalidomide and dexamethasone) (n=2), DVCd (daratumumab, bortezomib, cyclophosphamide and dexamethasone (n=2), DVMP (daratumumab, bortezomib, melphalan and prednisolone (n=1), DTd (daratumumab, thalidomide and dexamethasone (n=1) and DKd (daratumumab, carfilzomib and dexamethasone (n=1). Eleven patients (17.7%) received daratumumab monotherapy. The median duration of treatment with daratumumab was 12.3 months (range 0.5 to 39.4 months).

### Infusion-related reactions (IRRs)

***Pre-medications:*** All patients received corticosteroid pre-medication for the first dose of IV daratumumab: 20 were treated with methylprednisolone at a median dose of 100mg (range 40-100mg), 31 dexamethasone (17 at 20mg, 14 at 40mg; according to age and frailty); 11 methylprednisolone (range 40 - 100mg) in addition to dexamethasone (20mg or 40mg according to age and frailty). For non-corticosteroid prophylaxis, all (62 patients) received paracetamol, 10 received salbutamol nebulizer, 3 montelukast, 61 diphenhydramine and 2 oxymetazoline.

***Post-infusion prophylaxis:*** 24 of 62 patients received post-infusion corticosteroid prophylaxis, this being 12 who were given two days of prednisolone at 25mg/day, 5 receiving a median dose of 60mg methylprednisolone (range 20 - 100mg), 6 given 20mg dexamethasone, and information not known for 1 patient. Only 6 of the 62 patients received post-infusion non-corticosteroid prophylaxis (3 with salbutamol bronchodilator, 3 with diphenhydramine, and 2 with oxymetazoline).

***Infusion-related reactions (IRRs):*** 21 of 62 (33.9%) patients experienced infusion-related reactions (IRRs) after the first dose of IV daratumumab. Amongst these, 5 had CrCl <30mL/min, 8 had CrCl 30-60mL/min, and 8 had normal renal function (CrCl >60mL/min) (**Table 2**).

**Table 2.** Respiratory and Non-Respiratory Infusion Related Reactions (IRRs)

**Infusion related reactions (IRRs) – Respiratory**	**First Daratumumab Infusion (n=7)**	**Second Daratumumab Infusion (n=0)**
Grade 1	1	0
Grade 2	4	0
Grade 3	1	0
Grade 4	1	0
**Infusion related reactions (IRRs) – Non-Respiratory**	**(n=14)**	**(n=1)**
Grade 1	10	1
Grade 2	3	0
Grade 3	0	0
Grade 4	0	0
Unknown grade	1	NA
		

***Respiratory IRRs:*** 7 of the 21 IRRs were respiratory events, including bronchospasm, wheezing, dyspnoea and chest tightness. Two patients required additional treatment with bronchodilators (salbutamol), and 3 required additional antihistamines. One patient with grade-3 bronchospasm required admission to the intensive care unit, intubation and ventilation, and permanent discontinuation of daratumumab. Only 1 of the 7 patients who developed respiratory IRRs had a history of asthma. She received 40mg oral dexamethasone for pre-medication but not bronchodilator or montelukast. She developed grade-2 wheezing, which was resolved with salbutamol nebulizer treatment. Daratumumab was resumed with rate modification, and the first infusion dose was completed in 30 hours.

***Non-respiratory IRRs:*** 14 of the 21 patients experienced non-respiratory IRRs (5 with pyrexia, 4 with chills/rigors, 2 with hypotension, 1 with sinus tachycardia, and 2 with hives). Thirteen of the 14 patients experienced grades 1 - 2 reactions; there were no grades 3 or 4 reactions. Five patients were treated with antihistamines, 2 with corticosteroids, 4 with antipyretics, 2 with intravenous fluids. Twenty of the 21 patients who experienced IRRs resumed and completed daratumumab, all requiring rate modification. The median duration for 19 of the 20 patients (1 unknown) to complete the first dose of daratumumab was 12 hours (range 6 - 30 hours), compared with 7.8 hours (range 2 - 18 hours) for those without IRRs.

***Subsequent doses of IV daratumumab:*** One (1.6%) of 61 patients (who experienced grade-1 pyrexia with the first infusion of daratumumab and a total infusion duration of 10 hours), experienced a grade 1 IRR of pyrexia again with the second infusion of daratumumab (total infusion duration 8 hours). The remaining 60 patients experienced no IRRs with the second infusion. No patients experienced any IRRs with the third or subsequent doses of daratumumab.

***Adverse Events:*** 14 of 62 (22.6%) patients experienced treatment-emergent adverse events (TEAEs; **Table 3**).

**Table 3.** Treatment Emergent Adverse Events (TEAEs) and grade of events.

**Haematological Adverse Events (AEs)**	n=8	**Grade of Adverse Events**
Thrombocytopenia	2	Grade 2
Neutropenia	3	Grade 3 (n=2), Grade 4 (n=1)
Anaemia	3	Grade 2 (n=2), Grade 3 (n=1)
		
**Non-Haematological Adverse Events (AEs)**	n=6	**Grade of Adverse Events**
Pneumonia	4	Grade 2 (n=1), Grade 3 (n=2), Grade 4 (n=1)
Bacteraemia	1	Grade 3
Cardiovascular cerebral accident (CVA)	1	Grade 4
Upper respiratory tract infection	1	Grade 1
Hypokalaemia	1	Not known
Heart failure	1	Grade 3
Cellulitis	1	Grade 2

***Hematological Adverse Events (AEs):*** 8 of 62 (12.9%) patients experienced hematological AEs. Two patients had thrombocytopenias (both grade 2); three patients had neutropenia (2 had Grade 3, 1 patient treated with DRd therapy had grade 4 neutropenia but without sepsis), three patients had anemia (2 had grade 2, 1 had grade 3). All 8 patients received daratumumab combination therapy. The single patient with grade-2 thrombocytopenia required a short delay in the next dose of daratumumab. The cause of neutropenia in the two grade-3 patients was thought to be attributed to concurrent cyclophosphamide therapy and needed to receive G-CSF treatment. The 3 patients with anemia had chronic kidney disease (CKD) and received concomitant lenalidomide, contributing to their anemia.

***Non-Hematological Adverse Events (AEs):*** 6 of 62 (9.7%) patients experienced non-hematological AEs; 4 had pneumonia (one grade 2, two grade 3, and one grade 4), 1 had upper respiratory tract infection (grade 1), and 1 experienced hypokalemia. One myeloma patient with CrCl 35.7mL/min and treated with DRd regime experienced grade 4 (Nocardia) pneumonia and was admitted to ICU for intubation and ventilation. This occurred 14 months after the initiation of daratumumab, and the treatment regime DRd was permanently discontinued because the patient developed progressive disease and was no longer responding to treatment. One patient with AL amyloidosis and CrCl 32.3mL/min experienced grade-3 Klebsiella pneumonia with concomitant *Citrobacter bacteremia*, cerebral vascular accident (CVA) with hemorrhagic conversion (grade 4)) within 2 months from the start of daratumumab-based therapy (DRd). Another patient who had AL amyloidosis and myeloma with a CrCl of 26.9mL/min developed grade-2 pneumonia, grade-3 heart failure, and grade-2 lower limb cellulitis 5.8 months after the start of treatment with the DVd regime.

***Outcome:*** Only 1 patient with a TEAE discontinued daratumumab. No patients developed secondary malignancies during the follow-up period. At the time of data cut-off, 20 of the 62 patients were deceased: 18 from disease progression, 1 from (Nocardia pneumonia) infection (after having received 14 months of daratumumab before death). The cause of death of 1 patient was unknown.

## Discussion

This study demonstrated that daratumumab is tolerable to Asian patients, including those aged >75 years, low body weight, severe renal impairment, and chronic lung disease. The IRRs are generally limited to the first dose of the drug and are manageable (33.9% in our study) with mostly grades 1 or 2 reactions. IRRs are not more prevalent in patients with severe renal impairment and are comparable with clinical trial data (48% of patients experienced an IRR with the first infusion).^5^ Fewer than 23% of our patients experienced TEAEs, and most of them received combination therapy with lenalidomide, bortezomib, or cyclophosphamide.

One of the limitations of our study was the inability to capture all TEAEs, e.g., fatigue, nausea, and back pain, if they were not reported and documented in the medical notes. The median duration of follow-up of over 12 months, although it can be extended, is felt adequate to capture IRRs and TEAEs attributed to daratumumab. Adequate pre and post-medications, especially for the first dose of the IV infusion, is paramount to limit serious IRRs. Some patients in this study received IV methylprednisolone (in addition to oral dexamethasone) before the first dose of IV daratumumab. Many of them received a lower (20mg) dose of oral dexamethasone as part of their chemotherapy regime (e.g., DRd), and the practice amongst some clinicians was to administer additional IV methylprednisolone just for the first dose of daratumumab, to reduce the incidence of IRRs. We also routinely pre-medicate our patients with known chronic lung disease with salbutamol nebuliser, montelukast, and/or oxymetazoline.

For the first (and, in selected patients, the second) dose(s) of daratumumab, we routinely split the one-litre bag of reconstituted daratumumab into 2 separate bags. This has reduced drug wastage for patients who may require slow infusion rates due to IRRs. Administering the first dose of daratumumab over 2 separate days (8mg/kg per day) was also more tolerable for those with severe heart failure, a history of cardiac amyloidosis, and those with end-stage renal failure.

Subcutaneous (SC) daratumumab has been introduced in Southeast Asia recently, and is available as a fixed dose of solution for injection (1,800 mg/15 mL). It may be more suitable for those who cannot tolerate large volumes of intravenous fluids and for those with a significant history of drug allergies. Our experience is that SC daratumumab substantially reduces the chair time in our chemotherapy unit, resulting in reduced waiting time and improved patient satisfaction. It also leads to substantial cost savings and reduced length of hospital stay, as we routinely admit our patients for the first (and sometimes second) IV daratumumab infusion(s). However, SC daratumumab is not well studied in patients with low body weights (the median body weights of subjects in the 3 arms of the phase 2 PLEIADES study were 66kg, 77kg, and 80.6kg, respectively).^12^ Furthermore, based on our patient population (weight 35.2 – 83.2kg, median 58.7kg), it is more cost-effective for most patients with lower body weights to use intravenous over subcutaneous daratumumab. Therefore, IV daratumumab will likely continue to be used in parallel with SC daratumumab in many Asian countries unless the pricing strategy changes.

Although IV isatuximab is starting to be available in some Southeast Asian countries, it is only approved in combination with carfilzomib and dexamethasone (Kd), or pomalidomide and dexamethasone (Pd) in the relapsed/refractory setting. There is also little real-world data on the IRRs and TEAEs, especially amongst Asian patients. Furthermore, it would likely take years before a new agent would become affordable enough for routine clinical use. With the above considerations, cost containment measures, and increasing patient access, the anti-CD38 monoclonal antibody daratumumab will continue to be incorporated into the region’s standard treatment regimens for plasma cell dyscrasias.

### Ethics approval

The study was performed in line with the principles of the Declaration of Helsinki. Ethics approval was granted by the NHG (national healthcare group) DSRB (domain-specific review board) ethics committee (approval number: 2020/01305). The ethics committee waived informed consent due to the retrospective nature of the study.

### Authors’ contribution

All authors contributed to the study’s conception. Allison C.Y. Tso designed the study, wrote the manuscript, acquisition, analysis, and interpretation of the data. Samuel S.Y. Wang and Christian Gallardo collected the data. Dheepa Christopher and Ong Kiat Hoe critically reviewed the paper. All authors commented on previous versions and approved the final manuscript.
